# Clinical value and severity of myocardial perfusion defects in asymptomatic diabetic patients with negative or weakly positive exercise treadmill test

**DOI:** 10.7508/aojnmb.2013.01.004

**Published:** 2013

**Authors:** Seyed Rasoul Zakavi, Mehdi Taherpour, Zohreh Moossavi, Ramin Sadeghi, Vahidreza Dabbagh Kakhki, Haleh Rokni

**Affiliations:** 1 Nuclear Medicine Research Center, Mashhad University of Medical Sciences, Mashhad, Iran; 2 Cardiology Department, Razavi Hospital, Mashhad, Iran; 3 Endocrinology and Metabolism Research Center, Mashhad University of Medical Sciences, Mashhad, Iran

**Keywords:** Coronary artery disease, Diabetes mellitus, Gated myocardial perfusion SPECT, Silent Ischemia

## Abstract

**Objective:**

Although coronary artery disease (CAD) is the leading cause of death in type 2 diabetic patients, it is frequently asymptomatic. Myocardial perfusion imaging (MPI) is reported to show ischemia in a significant number of asymptomatic diabetic patients. We studied the prevalence and severity of myocardial perfusion defects in asymptomatic diabetic patients and its clinical impact.

**Methods and patients:**

One hundred thirty consecutive asymptomatic patients, aged 35-65 years with type 2 diabetes mellitus and with no history of CAD and no cardiac symptoms were recruited in the study. Echocardiography, electrocardiography (ECG), routine laboratory tests and exercise treadmill test (ETT) were performed and patients with weakly positive or negative ETT underwent Dipyridamole MPI. Patients with positive ETT were referred to coronary angiography. Patients were followed for at least 17 months (mean 21.7 months) and any cardiac event was recorded.

**Results:**

We studied 81 female and 49 male patients with mean age of 51.8 years. Negative, weakly positive and positive ETT result was noted in 74.3%, 15% and 10.7% respectively. 75% of patients with positive ETT had coronary artery disease in angiography. Gated myocardial perfusion SPECT was done in 106 patients. MPI showed reversible defect in 26.9% of the patients with a mean summed stress score of 3.3±1.8. Follow up completed in 112 patients and only one patient with abnormal MPI underwent coronary angiography followed by PTCA. No cardiac death, MI, UA or hospital admission occurred among our patients during follow up (17-26 months). Mean stress end diastolic volume (EDV) was significantly higher in patients with reversible defect compared to patients without reversible defect based on MPI findings (62.0±31.6 Vs 48.5±18.4 ml, P=0.04). Blood glucose and HbA1c were significantly higher in patients with ischemia compared to patients without ischemia (P<0.05). Meanwhile the ratio of TG to HDL was 6.06±3.2 in ischemic patients compared to 4.8±2.3 in normal subjects (P=0.03).

**Conclusion:**

Reversible defects are commonly seen in myocardial perfusion SPECT in asymptomatic diabetic patients and are mild in severity and not associated with adverse cardiac events. Routine approach for detection of CAD beginning with ETT seems to be appropriate in these patients.

## Introduction

Prevalence of Diabetes is increasing all over the world (1). It is estimated that more than 366 million people will have diabetes in year 2030 worldwide(1). The most common cause of death in type 2 diabetes patients is coronary artery disease(2). Coronary artery disease (CAD) is often asymptomatic in these patients until the onset of myocardial infarction or sudden cardiac death (3). Approach to detection of CAD is not uniform among physicians and multiple diagnostic techniques are suggested for this purpose including myocardial perfusion imaging (MPI), Exercise treadmill test (ETT), CT coronary angiography (CTCA), calcium scoring and stress echocardiography(4-7). Many studies showed silent ischemia in a significant number of asymptomatic diabetic patients(7-9). However the significance of detected ischemia in asymptomatic patients is not well known(9-11). Different guidelines suggested different approaches to detection of ischemia in diabetic patients(12-15). These guidelines are mainly based on the consensus opinion in the absence of valid clinical evidence. There is also a lot of controversy regarding treatment of asymptomatic diabetic patients with a ischemia detected by MPI(16-17). We studied asymptomatic diabetic patients using echocardiography, ETT and myocardial perfusion scintigraphy. We used routine approach for detection of ischemia beginning with ETT and proceeding to coronary angiography in case of positive ETT result. The remaining patients with negative or weakly positive ETT results may benefit from myocardial perfusion SPECT. In this study the prevalence of ischemia by MPI and its clinical impact in asymptomatic diabetic patients with negative or weakly positive ETT is explored.

## Methods and materials

We studied 130 consecutive patients with diabetes type 2 referred to endocrine clinic of Emam Reza Hospital. All patients aged 35-65 years with no history of coronary artery disease and no cardiac symptoms were recruited in the study. A written consent was signed by every individual who entered the study. A questionnaire was filled including patients demographic information and laboratory tests. Exclusion criteria were pregnancy, Q wave in electrocardiography (ECG), cardiomyopathy, cerebrovascular accident (CVA) or intermittent claudication, typical chest pain or chest pain equivalent, and history of myocardial infarction or coronary angioplasty. Laboratory tests including fasting blood sugar (FBS), 2hrs postprandial glucose, HbA1c, lipid profile and high sensitivity C reactive protein (hsCRP) were done in all patients. Then patients were referred to a cardiologist for echocardiography and exercise treadmill test. The exclusion criteria for ETT were diabetic foot, left bundle branch block (LBBB), proliferative retinopathy, severe degenerative knee joint disease and chronic kidney disease (CKD). If ETT was positive or strongly positive then coronary angiography was suggested to the patients. Otherwise patients were referred for gated myocardial perfusion SPECT (MPI). ETT was considered as weakly positive if there was horizontal or down sloped ST segment depression of 1-2 mm or up sloped ST depression of greater than 1.5 mm. It is considered as positive if ST segment depression was 2-3 mm and strongly positive if any of the followings was noted: ST segment depression > 3 mm anytime during exercise, > 1 mm during first stage of exercise, typical chest pain during stage 1, ST segment elevation, significant ventricular arrhythmia (3 or more consecutive PVCs), > 10 mmHg decrease in blood pressure. All ST segment changes were measured 80msec after J point in most reliable leads including V3-V6 and inferior leads.

Also patients who did not meet ETT inclusion criteria were studied by gated MPI. Dipyridamole gated myocardial perfusion SPECT was done in 106 patients using two day protocol. Dipyridamole was infused with a dose of 0.568mg/kg of body weight over 4 minutes and 740MBq of ^99m^Tc-MIBI was injected 2 minutes later. A dual head gamma camera (e-cam Siemens) was used for acquisition. All patients were imaged in supine position in a 180 degree arch from right anterior oblique (RAO45) to left posterior oblique (LPO45). If decreased uptake was noted in inferior wall in supine SPECT, imaging was repeated in prone position to exclude diaphragmatic attenuation. The images were reviewed by two experienced nuclear medicine specialist and consensus opinion was considered for interpretation. If consensus was not achieved, the opinion of the third nuclear medicine specialist was used for conclusion. Any reversible defect was considered abnormal. Any defect in inferior wall with improvement in prone images as well as any mild fixed anterior wall defect in women with normal wall motion was considered normal. Also semiquantification was done using a 17 segment model and 5 points grading score. In this system, the myocardium is reviewed in 16 short axis segments and one long axis segment. Normal uptake in each segment scored 0, mild decreased uptake 1, moderate decreased uptake 2, severe decreased uptake 3 and absent uptake scored 4. QPS software (Cedars-Sinai medical center, LA, USA) was used for semiquantification and comparison with normal data pool.

Patients with ischemia or infarction in myocardial SPECT as well as patients with coronary stenosis on angiography were referred to cardiologist for appropriate therapy. All patients in our study were treated during the follow up according to the AHA/ACC guidelines(14). Patients were followed every 6 months for at least 17 months and any cardiac event, admission to cardiac unit, angioplasty or cardiac surgery was recorded. The project was approved by a local ethical committee.

Univariate analysis was done for description of the data. Statistical analysis was performed using SPSS software (SPSS, V 11.5) to compare different variables in patients with and without ischemia using independent t- test and chi-square tests. P value of <0.05 was considered significant in all comparisons.

## Results

One hundred and thirty patients (81 female, 49 male) with mean age of 51.8 ±7.3 years enrolled in the study. The mean duration of diabetes since diagnosis was 7.8 ±6.3 years. The mean body mass index (BMI) was 27.9±3.8 kg/m^2^. Table 1 shows demographic and laboratory findings in our patients. Echocardiography was done in all patients by a single instrument (Vivid 3, GE, Norway). The echo findings showed mean LV ejection fraction of 61.9±5.7%. Left ventricular motion abnormality was noted in 3.1% and Left ventricular hypertrophy was seen in 55 patients (43%). Rest ECG showed mild ST depression or T inversion in 14 patients (10.8%).

**Table 1:**
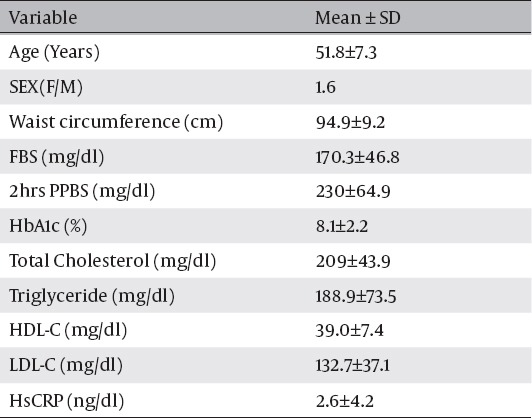
Demographic and Laboratory information of patients.

ETT was done in one hundred and thirteen patients of which 74.3% were negative, 15% were weakly positive, 1.8% were positive, and 8.9% were strongly positive. Maximum Heart Rate (MHR) was defined as 220 minus age and all patients undergoing ETT reached at least 85% of MHR.

Of 130 patients, 10 patients didn’t agree to undergo myocardial perfusion imaging. Twelve patients had positive or strongly positive ETT and were directly referred to coronary angiography. Gated myocardial perfusion SPECT was done in 106 patients. In 2 patients nongated imaging was done due to rhythm irregularities. Seventy nine patients (73.1%) had normal MPI, while 29 patients (26.9%) had reversible defects. No Fixed defect was noted in our patient. Mean (±SD) summed stress score was 3.3 ±1.8 and mean (SD) summed rest score was 0.72±0.75 in patients with reversible defect. Mean (SD) stress end diastolic volume (EDV) was significantly higher in patients with reversible defect compared to patients without reversible defect based on MPI findings (62.0±31.6 Vs 48.5±18.4 ml, P=0.04). Interestingly the rest EDV was higher too in patients with reversible defects, however it did not reach statistical significance (P=0.07).

Angiography was recommended for 12 patients after a positive or strongly positive ETT and was performed in 4 patients. One patient had 3 vessel disease and underwent coronary artery bypass grafting (CABG). Another patient had single vessel disease and underwent balloon angioplasty and a drug elutant stent was placed in the LAD. One patient had severe 3VD and did not accept CABG and is under medical treatment with no cardiac event up to 24 months. The 4^th^ patient who underwent coronary angiography had a 50% stenosis in LAD and is being treated with medical therapy.

Patients were visited every 6 months and any cardiac event was recorded. Also patients were called to come for review of data and visit if they were not visited in due time. One hundred and thirteen patients (86.9%) completed follow up. Patients were followed for at least 17 months and mean time of follow up was 21.7±1.7 months. During follow up 1 patient with abnormal MPI underwent coronary angiography followed by PTCA. No cardiac death, myocardial infarction (MI), unstable angina (UA) or cardiac admission was occurred among our patients.

Mean age of patients with and without reversible defect was 51.95±7.3 and 51.97±7.0 years respectively. The mean follow up time was not significantly different between patients with normal MPI (22.1±1.7 months) and patients with reversible defect (21.2±1.1 months).

Blood glucose and HbA1c were significantly higher in patients with ischemia compared to patients without ischemia (P<0.05). However there was no significant difference in total cholesterol, HDL-C and LDL-C, triglyceride(TG) or HsCRP level in patients with ischemia compared to patients without ischemia(Table 2). Meanwhile the ratio of TG to HDL was 6.06±3.2 in ischemic patients compared to 4.8±2.3 in normal subjects (P=0.03).

**Table 2:**
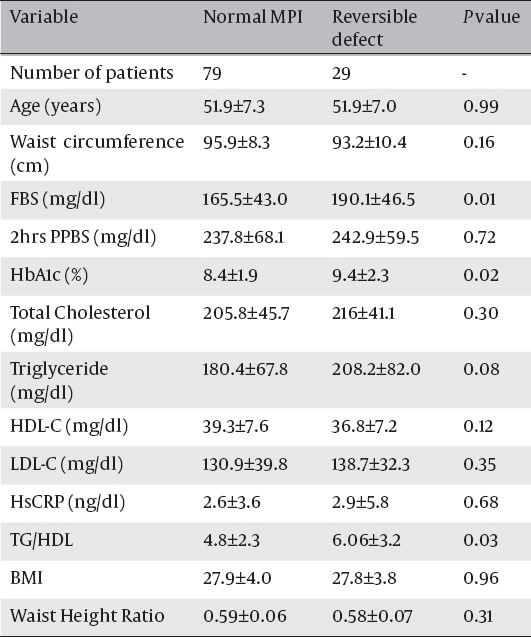
Comparison of laboratory findings in patients with and without reversible defect in MPI.

Waist circumference, BMI and waist to height ratio (WHR) were not significantly different between patients with and without ischemia in myocardial perfusion SPECT (P=0.3).

Using chi-square test, prevalence of risk factors was compared in patients with and without reversible defects (Table 3).

**Table 3:**
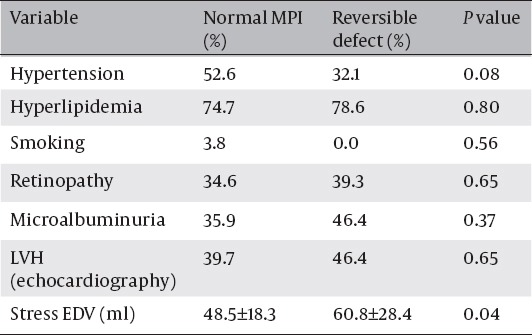
Prevalence of traditional risk factors in patients with and without myocardial perfusion defect.

There was no significant difference in prevalence of hypertension, hyperlipidemia, smoking, family History of CAD, microalbuminuria, retinopathy or foot ulcer in patients with and without reversible defect(P>0.05).

## Discussion

More than 200 million people in the world suffer from diabetes and 65%-85% of these patients finally die from CAD(1). Myocardial infarction and sudden cardiac death are common presentation of CAD in diabetic patients(3). Also the number of patients with diabetes mellitus was increasing among patients admitted to the hospital with myocardial infarction(18). In addition diabetic patients with abnormal SPECT or abnormal stress echocardiography had worse prognosis than nondiabetic patients(19). So many researchers tried to detect silent ischemia in asymptomatic diabetic patients in order to prevent future cardiac events(7-8, 10, 20-22). Variable modalities are used for detection of CAD in asymptomatic diabetic patients with different success rates. These techniques include CT-Angiography and calcium scoring, MPI, stress echocardiography and ETT(4-7, 23-24). However the clinical benefit of screening of these patients remains to be proved(4).

We used a routine approach for detection of CAD using ETT first. Our study showed that 10.7% of patients had positive or strongly positive ETT. From these patients who accepted coronary angiography 75% had significant CAD and 25% had borderline stenosis of about 50% in coronary arteries.

Also reversible myocardial perfusion defect was observed in 29 patients (26.9%). This is similar to the findings by DIAD study which reported 22% of ischemia among asymptomatic patients (7). In DIAD study the age range was 50-75 years while our study had a wider range of age(7). Sozzi et al. found 28% prevalence of ischemia in a group of asymptomatic diabetic patients who had a mean age of 62 years (24). A recent study showed a prevalence of 25% of ischemia in MPI in asymptomatic diabetic patients with mean age of 53±10 years(25). Similar findings in these studies may suggest age independence of prevalence of silent ischemia in diabetic patients.

From 29 patients with silent ischemia in our study only one patient (3.2%) underwent PTCA during follow up. No other minor or major cardiac event was noted in this group. Young et al. also reported PPV of 6% for reversible defects in predicting MI or cardiac death during a mean follow up of 4.8 years in asymptomatic patients with diabetes(2). In a randomized trial they noted that use of MPI had no discernable effect on subsequent cardiac events(2).

In our study mean summed stress score was 3.3 ±1.8 in patients with reversible defect suggesting that most of these patients had minimal ischemia. Our study also showed that only 2.7% of asymptomatic diabetic patients had defects involving more than 10% of the left ventricle. In DIAD study the large defect (≥10%) was reported in only 1% of population. It is reported that the larger the MPI defect in asymptomatic diabetic patients, the greater the incidence of cardiac events(2). Also in asymptomatic patients (not all diabetics) MPI ischemia of ≥7.5% (using 20 segment analysis) was considered as independent predictor of future events(11). The severity and extent of the ischemia as determined by SSS is not extensively studied in asymptomatic diabetic patients. Our study showed that MPI defects in asymptomatic diabetic patients is mainly small in magnitude (as assessed by SSS) and does not result in adverse cardiac events.

Functional indices of the LV using MPI have been studied in asymptomatic diabetic patients(26). In one study 16.7% of patients had low LV ejection fraction (LVEF) which was associated with older age and higher annual mortality rate(26). No patient in our study had LVEF of less than 45% in gated myocardial perfusion SPECT. However mean post stress EDV was significantly higher in patients with reversible defects compared to patients without reversible defects. Also left ventricular hypertrophy was seen in 43% of our patients. Left ventricular hypertrophy is reported to be higher among diabetic patients compared to nondiabetic subjects(27). Bertoni et al. in a study of nearly 5000 diabetic patients by MRI found significant ethnic-specific differences in LV mass as well as EDV and concluded that these findings are not related to the subclinical CAD(27).

In our study FBS and HbA1c level was higher in patients with ischemia confirming that ischemia is more frequent in poorly controlled patients. It is also reported that higher glucose level in these patients may be associated with higher complications(3). However we could not find any difference in other laboratory variables like total, HDL and LDL cholesterol, triglyceride, HsCRP and 2hrs postprandial blood glucose level between patients with and without ischemia.

Bittner et al(28) reported that the ratio of TG/HDL is a powerful independent predictor of all cause mortality and cardiovascular events in women. In our study TG/HDL ratio was significantly higher in patients with ischemia compared to normal subjects.

Tseng in a study of diabetic patients found that among obesity factors of BMI, waist circumference, waist to hip ratio and waist to height ratio, the waist to height ratio had the greatest magnitude of odds ratio between patients with and without CAD(29). However others could not find significant difference in obesity indices and coronary artery calcifications(30). Also it is shown that intima-media thickness of the carotid is not significantly different in patients with and without central obesity(31). We also could not find any difference in BMI, waist circumference and waist to height ratio between patients with and without ischemia.

As intensive cardiac risk factor modification is recommended in all patients with type 2 diabetes, the role of screening tools for detection of mild ischemia is also questioned(32). Routine use of MPI or stress echocardiography in asymptomatic diabetic patients as well as its cost effectiveness is challenged by many authors(33-36). MPI may detect clinically nonsignificant ischemia in these patients and may not change the clinical decision(2, 35). However, if MPI findings showed high risk pattern, the patients get a survival advantage by undergoing CABG (37). No survival advantage was noted in treating patients with low to intermediate risk MPI patterns(37). In this study we found that MPI defects are mainly small in magnitude in asymptomatic diabetic patients with negative or weakly positive ETT.

Our study had a limitation of relatively low number of patients. Longer follow up in a large number of patients and analysis in different subgroups as well as cost effectiveness analysis will help in defining the best approach to CAD detection in asymptomatic diabetic patients.

## Conclusion

Our study showed that although myocardial perfusion defects were frequently seen in asymptomatic diabetic patients, defects were of small magnitude and did not reflect adverse outcome. Routine MPI imaging in all asymptomatic diabetic patients is not recommended. Routine approach to CAD beginning with ETT may be more justifiable in asymptomatic diabetic patients.
